# Commentary: Intersectional perspectives on COVID-19 exposure

**DOI:** 10.1038/s41370-021-00336-2

**Published:** 2021-05-08

**Authors:** Jade S. Sasser, Bronwyn Leebaw, Cesunica Ivey, Brandon Brown, Chikako Takeshita, Alexander Nguyen

**Affiliations:** 1 Department of Gender & Sexuality Studies, University of California, Riverside, CA USA; 2 Department of Political Science, University of California, Riverside, CA USA; 3 Chemical and Environmental Engineering, Center for Environmental Research and Technology, University of California, Riverside, CA USA; 4 School of Medicine, University of California, Riverside, CA USA

Last November, the American Medical Association released a public statement [1] to announce that the organization now recognizes racism as a public health threat and commits to actively working to dismantle racist policies and practices that affect health care. Local and state leaders have made similar declarations in recent months, in response to a growing awareness of the inequities associated with disparate exposures, severe illness and death due to COVID-19 in communities of color. Such statements call for urgent attention to the health effects of structural injustice and its disparate impacts on human health.

This commentary calls for analyses of the intersecting patterns of inequality and systemic logics that may not be obvious to leaders and policymakers. Specifically, we highlight the need for an intersectional approach, one that addresses the compounding effects of overlapping and interdependent systems of oppression [2], in this case, race and class. This is vital to addressing the health effects of structural and systemic racism and class inequality that are often rendered invisible due to either lack of attention, or narrow perspectives that study these factors in isolation. We make the case for intersectional approaches to investigating the systemic health inequities that intensify vulnerabilities associated with COVID-19 infection, drawing on inland California as a case study.

Public health scholars were among the first to sound the alarm about the health inequities associated with COVID-19. An early study of the pandemic found that U.S. counties with higher Black populations had higher comorbidities, higher proportions of uninsured people, as well as higher proportions of unemployed people, higher exposures to air pollution, and more COVID-19 diagnoses and deaths [3]. A similar study of Latino-majority U.S. counties found consistently higher COVID-19 incidence, despite no significant differences in underlying health conditions or social distancing scores compared to other counties [4]. We cannot understand these factors through the lens of race alone, in part because both race and low socioeconomic status (SES) are closely linked to the likelihood of being a member of one of the most vulnerable classes in the pandemic: an essential worker. “Essential workers” is a classification that varies from state to state, but generally encompasses work deemed necessary to maintain critical infrastructure, associated with transportation, health care, and the supply of food and energy. These workplaces have been central sites of infection and transmission of COVID-19 [5]. Further, essential workers are more likely to live in multi-family homes, which magnifies vulnerabilities encountered in the workplace [6].

Research demonstrates that people of color in the U.S. are more likely to be essential workers. For example, Latino and Black workers are employed in warehousing at twice the rate of all other industries. Latino workers compose the largest single race/ethnic group in front-line warehousing jobs, while Black workers make up one-quarter of the workforce in both warehousing and e-commerce. Overall, workers of color constitute 66% of warehousing industry workers and 55% of workers in e-commerce, even though workers of color account for just 37% of the total U.S. labor force [7].

Warehouse workers, along with their families, are vulnerable to health effects associated with exposure of air pollution caused by the expansion of warehouses in their communities as warehousing facilities increase the flow of diesel-fueled freight trucks into the local communities where workers and their families typically reside [8]. Further, the risks of COVID infection are also exacerbated by pre-existing vulnerabilities that have been linked with exposure to air pollution. A nationwide study showed that just a small increase in fine particulate matter (PM_2.5_), was associated with an 11% increase in the COVID-19 death rate for U.S. counties [9], accounting for the confounding effects of age, race, socioeconomic status, behavioral risk factors, etc. In international studies, air pollution is also cited as an important factor for determining morbidity or mortality risk during the COVID-19 pandemic [10, 11].

People of color and those with a lower income have a higher exposure to air pollution, particularly in the inland region of Southern California, in which Riverside and San Bernardino Counties have disproportionately high populations of color as well as high poverty rates. Significantly, the Amazon Fulfillment Centers, which are among the top employers in the region, are in and adjacent to Ward 1, where 77% of the population identified as Hispanic origin, and where poverty and asthma rates are among the highest in California [12]. Diesel trucks carrying cargo for the fulfillment centers flood the freeways that traverse the region.

**Fig. 1:**
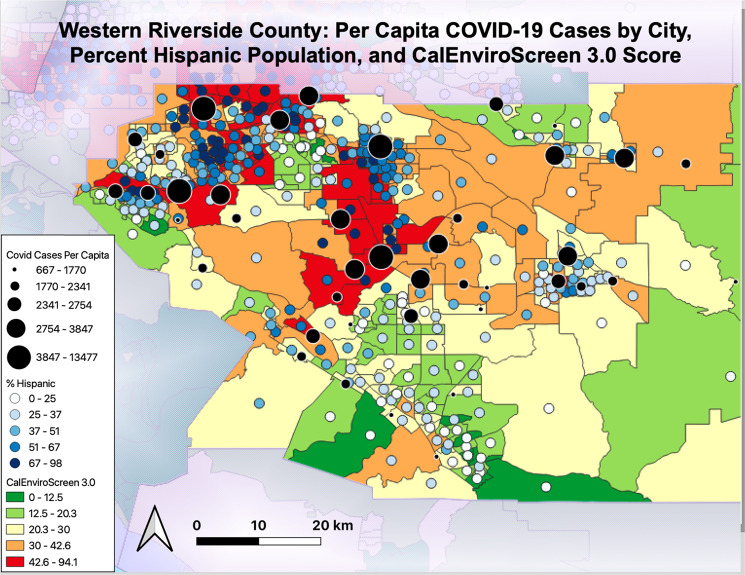
Western Riverside County: Per Capita Covid-19 Cases by City, Percent Hispanic Population, and CalEnviroScreen 3.0 Score. Map of per capita COVID-19 cases as of November 20, 2020, overlaid with Census tract data for percent Hispanic Population and CalEnviroScreen 3.0 score, where higher values denote greater environmental vulnerability.

Related to this truck travel, Riverside County has received a grade of “F” in the American Lung Association State of the Air report for the past two years (2019 and 2020) [13]. The inland region of Southern California is burdened with emissions from the Los Angeles city center, as well as local source emissions, which form secondary pollution under the intense California sun. Warehouses in Southern California are disproportionately sited in Black and Latino communities, resulting in higher levels of pollution associated with diesel fueled trucks, increasing health risks associated with proximity to traffic. An examination of environmental vulnerability (quantified by California Office of Environmental Health Hazard Assessment) and per capita COVID-19 cases reported by the Riverside University Health System demonstrates the same pattern of disproportionate COVID-19 cases, ethnicity, and environmental vulnerability in Western Riverside County (Fig. 1). The highest per capita cases occurred in communities with large Hispanic population and/or higher environmental vulnerability in most cases.

Given these intersecting vulnerabilities, an overarching question is how to allocate resources equitably in the COVID-19 pandemic and other epidemics. How can an intersectional analysis best address the ways structural inequality and unequal healthcare access shape exposures for marginalized people? How can researchers best work across disciplines to address these intersectional linkages? What studies are most informative for supporting inclusive and sustainable solutions to highly intersectional problems?

We call for research that provides an understanding of how risk factors including air pollution exposure, working conditions, race, and SES interact, in order to better understand how interventions to reduce COVID-19 exposure should take place. Furthermore, we highlight the need to advocate for regulatory infrastructure that maintains safety protocols in workplaces that disproportionately expose communities of color and the poor to COVID-19. Finally, we recommend that environmental health scientists take further steps to characterize the exposome in homes and workplaces of the most severely impacted populations during the COVID-19 crisis (e.g., essential workers), and build databases of exposure data at the individual level that can be mined for predictive and mitigation purposes [14]. Only then will we be able to accurately characterize links between air pollution exposure and health risks for highly susceptible populations [15].
